# Methoprene-Tolerant (Met) Is Indispensable for Larval Metamorphosis and Female Reproduction in the Cotton Bollworm *Helicoverpa armigera*

**DOI:** 10.3389/fphys.2018.01601

**Published:** 2018-11-15

**Authors:** Long Ma, Wanna Zhang, Chen Liu, Lin Chen, Yang Xu, Haijun Xiao, Gemei Liang

**Affiliations:** ^1^Jiangxi Key Laboratory of Bioprocess Engineering, College of Life Sciences, Jiangxi Science and Technology Normal University, Nanchang, China; ^2^Institute of Entomology, Jiangxi Agricultural University, Nanchang, China; ^3^State Key Laboratory for Biology of Plant Diseases and Insect Pests, Institute of Plant Protection, Chinese Academy of Agricultural Sciences, Beijing, China

**Keywords:** *Helicoverpa armigera*, methoprene-tolerant, juvenile hormone, vitellogenesis, reproduction

## Abstract

Juvenile hormone (JH) represses larval metamorphosis and induces adult reproduction in insects. Methoprene-tolerant (Met) is identified as an intranuclear receptor that mediates JH actions. In the present study, we characterized a Met from the severe agricultural pest, *Helicoverpa armigera*, namely HaMet. In the larval stage, *HaMet* is predominantly expressed in the epidermis and midgut, and is upregulated before each molting, whereas in adults *HaMet* is maximally expressed in the ovary, testis, and fat body. The immunofluorescence assay revealed that HaMet was distributed in the longitudinal and circular muscle layers of midgut in larvae, whereas in the ovary of female adults, HaMet was localized in the nucleus of the oolemma. Knockdown of *HaMet* in final-instar larvae shortened the time of pupation, induced abnormal pupation, and dampened pupation rate. In female adults, *HaMet* depletion severely suppressed the transcription of *Vitellogenin* (*Vg*) and *Vitellogenin Receptor* (*VgR*), disrupted the Vg accumulation in fat body and the yolk protein uptake in oocytes, and finally led to an impaired fecundity. Our findings therefore confirmed that HaMet acted as a nuclear receptor of JH and played an essential role in larval metamorphosis, vitellogenesis, and oocyte maturation.

## Introduction

Juvenile hormones (JHs) are sesquiterpenoid compounds that are synthesized and secreted by corpora allata. In insects, JHs play crucial roles in controlling metamorphosis, reproductive development, and some aspects of mating behavior in both male and female insects (Riddiford, 2008). In the larval stage, high titer of JH maintains larval molting and prevents metamorphosis, whereas a drop or absence of JH titer in the final instar allows insect metamorphosis with the rise of 20-hydroxyecdysone (20E) levels (Nijhout, 1994). Being an important hormone-regulating insect development, 20E has been well characterized by later studies (Siaussat et al., 2007; Jing et al., 2016). Conversely, the molecular mechanism addressing the action of JH has remained enigmatic until recently, when the transcription factor methoprene-tolerant (Met) was identified as the potential JH receptor (Jindra et al., 2013).

Methoprene-tolerant protein has a typical basic helix-loop-helix Per-Arnt-Sim (bHLH-PAS) domain. This protein is found to be capable of binding to both natural JH-III and a mimicker of JH (methoprene) with high affinity (Ashok et al., 1998; Miura et al., 2005; Charles et al., 2011). Since the first characterization of Met in *D. melanogaster* (Wilson and Fabian, 1986), homologs of Met have been identified from a broad range of insect species, including *Aedes aegypti* (Zhu et al., 2010), *Tribolium castaneum* (Konopova and Jindra, 2007), *Bombyx mori* (Kayukawa et al., 2012), *Pyrrhocoris apterus* (Konopova et al., 2011), and *Nilaparvata lugens* (Lin et al., 2015). Accumulating evidences suggest that the anti-metamorphic function of JH is correlated with Met. In the beetle *T. castaneum*, knocking down *TcMet* disrupted larval-pupal ecdysis and induced precocious adult development upon partial ecdysis, suggesting that Met was involved in anti-metamorphic JH signal transduction (Konopova and Jindra, 2007). In *P. apterus*, silencing *Met* at the penultimate-instar nymphs caused the development of adult features instead of molting to the final nymphal instar (Konopova et al., 2011). Moreover, studies in *A. aegypti*, *T. castaneum*, and *B. mori* have revealed that the heterodimerization of Met with a steroid receptor coactivator was required for the JH-induced transcription of JH target genes (Li et al., 2011; Zhang et al., 2011). Thus, Met was regarded as the JH intranuclear receptor (Jindra et al., 2013). Subsequently, Krüppel-homolog 1 (Kr-h1), a transcription factor with a DNA-binding domain consisting of eight zinc fingers, was reported to work on the downstream of Met in the JH signal pathway (Belles and Santos, 2014).

Vitellogenesis, a key process in female reproduction in insects (Sappington and Raikhel, 1998), is under the control of JH and/or ecdysone. These two hormones are the main inducers of vitellogenin (Vg) synthesis from the fat body and Vg uptake into the developing oocyte via vitellogenin receptor (VgR)-mediated endocytosis (Raikhel et al., 2005; Roy et al., 2018). Recent studies in mosquitos and locusts have revealed that JH regulated the expression of Vg based on its receptor Met (Zou et al., 2013; Guo et al., 2014). In these insects, Met is indispensable for egg production and *Vg* expression, and knockdown of Met resulted in the retardation of ovarian development (Zou et al., 2013) and the lower egg production (Li et al., 2011). Further study in the cockroach, *Diploptera punctata*, showed that knockdown of *Met* resulted in an arrest of oocyte development and *Vg* gene expression (Marchal et al., 2014). The similar function of Met in reproduction was detected in *T. castaneum* (Minakuchi et al., 2009), *A. aegypti* (Shin et al., 2012), and *P. apterus* (Konopova et al., 2011).

Methoprene-tolerant is undoubtedly an important transcription factor playing a crucial part in insect development and reproduction. However, the function and mechanism of Met regulation is not well understood owing to its complex roles in the JH pathway. In the present study, we obtained a full-length *Met* from the cotton bollworm, *Helicoverpa armigera* (*HaMet*). The expression patterns of *HaMet* were first investigated. An immunofluorescence assay was performed to determine the distribution of HaMet protein in the larval midgut and the adult ovary. Furthermore, the functions of HaMet in larvae-pupae transition and female reproduction were investigated by RNA interference (RNAi). Our study provides new insights into how Met functions in larval metamorphosis and female reproduction.

## Materials and Methods

### Insects and Tissues Sampling

The *H. armigera* used in this study were reared in the laboratory within a controlled environment of 27 ± 2°C, 75 ± 10% RH, and a photoperiod of 14: 10 h (L:D). The *H. armigera* strain was divided into five instars according to their ecdysis times. The larvae were first reared on an artificial diet in the 24-well plate, and then transferred into 25 ml glass tubes containing an artificial diet at the 5th instar for pupation (one larva per tube). After emergence, the adults were placed in cages (30 cm × 60 cm × 30 cm) for oviposition and supplied with 10% sugar solution.

To examine the developmental expression profiles of *HaMet*, individuals were collected from egg, larvae (1st, 2nd, 3rd, 4th, and 5th instar), pupae, and adults. Samples were prepared at intervals of 1 day during larval stages, 2 days from pupation to adult emergence, and 2 days for adults. For tissue expression analysis, tissues (including head, epidermis, midgut, hemolymph, and fat body) were dissected from the 5th-instar larvae, and tissues from adults (including ovary, testis, head, epidermis, midgut, hemolymph, and fat body) were stripped in phosphate-buffered saline (PBS). All the samples were frozen immediately in liquid nitrogen and stored at -80°C until RNA isolation. Four biological replicates containing three to 50 individuals were prepared for each experiment.

### RNA Isolation and cDNA Synthesis

Total RNA was extracted using Trizol reagent (Invitrogen, Carlsbad, CA, United States). The integrity of each RNA sample was checked with 1% agarose gel electrophoresis, and the RNA quantity was determined using a NanoVue spectrophotometer (GE-Healthcare, Germany). After digestion of residual genomic DNA with DNase I (Promega, Madison, United States), 2 μg total RNA was reverse transcribed in 20 μl reaction mixtures using the Fast Quant RT kit (TIANGEN, Beijing, China) according to the manufacturer’s instruction. The synthesized first-strand cDNAs were stored at -20°C.

### Molecular Cloning and Bioinformatics Analysis

A 768 bp cDNA fragment encoding the partial of *HaMet* was first amplified, and the rapid amplification of cDNA ends (RACE) was used to obtain the full-length cDNA. In brief, the 3′- and 5′-RACE cDNA templates were synthesized using the SMART RACE cDNA amplification kit (Clontech, Mountain View, CA, United States). Gene-specific primers (GSP) were designed on the basis of the *HaMet* fragment. The PCR amplification was conducted by means of touchdown with the GSP and the universal primer mixture (UPM). Then the 5′- and 3′-RACE products were purified and sequenced. After sequence splicing, the open reading frame (ORF) of *HaMet* was further confirmed by PCR amplification.

The BLASTx algorithm was employed to run the similarity searches. The tools available in the ExPASy proteomics server^1^ were used to determine the putative molecular weight and isoelectric point. Moreover, the SMART program^2^ was used to identify the conserved domains. The percent identity of the amino acid sequences was calculated from single pairwise alignments using Vector NTI. Finally, a phylogenetic tree was constructed with MEGA 7.1 using the neighbor-joining method with a p-distance model and a pairwise deletion of gaps. Bootstrap values of tree branches were assessed by resampling amino acid positions 1000 times.

### Analysis of *HaMet* Expression by qRT-PCR

The qRT-PCR analysis was performed using SuperReal PreMix Plus (SYBR Green) (Tiangen Biotech, Beijing, China) on ABI 7500 Fast Real-Time system. Each reaction was performed in a 20 μl volume containing 1 μl of cDNA, 10 μl of SuperReal PreMix (2×), 0.6 μl of each primer (10 μM), 0.4 μl of ROX Reference Dye (50×), and 7.4 μl of ddH_2_O. The qRT-PCR program consisted of one cycle of 95°C for 1 min, followed by 40 cycles of 95°C for 5 sec, 60°C for 15 sec, and a melt curve stage. Two house-keeping genes, *Ef-a* (accession no. XM_021329970) and *ß-actin* (accession no. EU527017), were used as reference genes to normalize the target gene expression and to correct for sample-to-sample variation. The comparative 2^-ΔΔ*Ct*^ method was used for the normalization of gene expression. To ensure reliability, each reaction for each sample was performed in triplicate with four biological replicates. Negative control without template was included in each reaction. The primer sequences of the genes were listed in Supplementary Table S1.

### Immunofluorescence Staining of HaMet

The midgut from 5th-instar larvae and the ovary tubules from 2-day-old female adults were dissected, respectively, and prefixed in 4% paraformaldehyde for 30 min at 4°C, followed by infusion overnight in a solution of 20% sucrose in 0.1 M PBS plus 0.1% Triton X-100 (PBST) at 4°C. The tissues were then embedded in O.C.T. compound (optimum cutting temperature compound, Sakura, United States). Ultrathin sections of 12 μm thickness were cut, gathered on Super Frost Plus slides (Menzel-Gläser, Braunschweig, Germany), and dried at room temperature for 1 h. These sections were post-fixed in 4% paraformaldehyde for 30 min at 4°C. The sections were then rinsed thrice in PBST, and blocked with 5% normal goat serum (NGS)-PBST at room temperature for 1h. After washing thrice with PBST, the slides were incubated at 4°C overnight with HaMet antiserum (kindly provided by Pro. XiaoFan Zhao, Shandong University) diluted at 1:1000 in 5% NGS. The sides were then rinsed thrice in PBST, incubated for 2 h at room temperature with goat anti-rabbit Alexa Fluor 488 dye (1:500 in TNB Buffer), and treated with DAPI (Beyotime Biotechnology, China) at room temperature for 10 min. Finally, the sections were observed with a confocal laser scanning microscope (Zeiss, Oberkochen, Germany) after washing with PBST thrice for 10 min each.

### RNAi Experiment

Double-stranded RNA (dsRNA) was prepared using a HiScribeTM T7 Transcription Kit (New England BioLabs, Ipswich, MA, United States) from the PMD18 plasmid (Takara, Dalian, China) containing a 710 bp fragment of HaMet (from 872 to 1581), and the synthesized dsRNA was injected into larvae or female adults as described previously (Zhang et al., 2016b). A segment of *GFP* (green fluorescent protein) was used to produce dsRNA for *GFP* (ds*GFP*) as control.

To investigate the function of HaMet in larval development, 1 μl solution (3 μg/μl) of dsRNA targeting *HaMet* (ds*HaMet*) was injected into the abdomen of 5th-instar larva. The control individuals were treated with the same dose of ds*GFP*. Each treatment contained 72 individuals, then five individuals were randomly selected for qRT-PCR analysis at every 24 h after injection and the pupation time (from 5th instar 0 h to pupa) and the pupation rate of the remaining samples were recorded.

To examine the effect of *HaMet* depletion on fecundity, newly emerged female adults were injected in the abdomen with ds*HaMet* (5 μg), and the controls were injected with an equivalent dose of ds*GFP*. The injection point was sealed with geoline immediately. Subsequently, ten individuals were randomly selected at 24 h, 48 h, 72 h, and 96 h after injection, respectively; then the tissues of fat body and ovary were dissected to investigate the RNAi efficiency. In addition, in each treatment, the ovarian phenotypes of female moths were observed. In brief, the ovaries from dsRNA-treated individuals (3 days after treatment) were dissected in PBS and photographed with a stereomicroscope (Olympus SZX16, Tokyo, Japan), and the numbers of follicles at different developmental stages were recorded as described previously (Zhang et al., 2015). The observations were conducted for 15 females in each treatment. Meanwhile, 50 treated females were chosen for an oviposition bioassay. Each female was paired with two untreated males in one plastic cup (8 cm in diameter, 10 cm high). The plastic cups covered with one layer of 10 cm × 10 cm gauze were kept under the same condition as mentioned. The cotton wicks were placed on the gauze to supply 10% sugar solution, and both the gauze and the cotton wick were changed daily to count the number of eggs laid. All the experiments were performed thrice.

### Hormone Treatments

For hormone mimic treatments, methoprene (Sigma-Aldrich, St. Louis, United States) and 20E (Sigma-Aldrich, St. Louis, United States) were dissolved in acetone. 20E or methoprene was topically applied to the dorsal abdomen of 5th-instar larvae (2 μg/larva), and controls were treated with the same dose of acetone.

When RNAi and JH mimic treatments were to be combined, newly emerged moths after dsRNA treatment were further treated with methoprene (5 μg per moth) 1 day after the dsRNA treatment. At 2 days after the methoprene application, the tissue of fat body was dissected and subjected to qRT-PCR and western blot analysis.

### Western Blot Analysis

The tissue of fat body was homogenized in lysis buffer (8 M urea, 4% chaps, 40 mM Tris–HCl, 5 mM EDTA, 1 mM PMSF and 10 mM DTT, pH 8.0) containing a mixture of protease inhibitors (Roche, Switzerland). The concentration of crude protein was determined by a Bio-Rad protein assay using bovine serum albumin (BSA) as the standard. The samples were then diluted with loading buffer to obtain an equal amount of total protein. After the proteins were separated by 12% (w/v) SDS–PAGE, the samples were transferred onto nitrocellulose (NC) membrane blotting filters at 100 V for 1 h at 4°C. The membranes were then blocked with 5% (w/v) skimmed milk in PBST at 4°C overnight. After washing thrice with PBST, the blocked membrane was incubated with *H. armigera* β-actin antibody (1:2000 dilution) and HaVg antibody (1: 4000 dilution) (Zhang et al., 2016b) for 1 h at room temperature. After three washes with PBST, the membrane was incubated for 1 h at room temperature with goat anti-rabbit IgG HRP-linked secondary antibody (Sigma, St. Louis, United States) at 1:10,000 dilution with PBST. The immunoreactivity was visualized using an enhanced electrochemiluminescence detection kit (TransGen, Beijing, China) and photographed by Image Quant LAS4000 mini (GE-Healthcare, Germany).

### Data Analysis

All the data in this study were presented as means ± SE. Significant differences were determined by Student’s *t*-test or one-way analysis of variance (ANOVA) followed by a least significant difference test (LSD) for mean comparison. All statistical analyses were performed with SAS 9.20 software (SAS Institute, Cary, NC, United States).

## Results

### Cloning and Sequence Analysis of *HaMet*

The 5′- and 3′-end sequences of *HaMet* were obtained using the RACE technology. After assembling, a full-length transcript encoding *HaMet* was obtained (Accession no. KJ825895). This transcript is 2511 bp, including a 351 bp 5′-untranslated region (UTR), a 579 bp 3′-UTR, and 1581 bp ORF. The latter encodes a 526-amino acid protein with a predicted molecular mass of 59.88 kDa and an estimated isoelectric point of 7.1.

**FIGURE 1:**
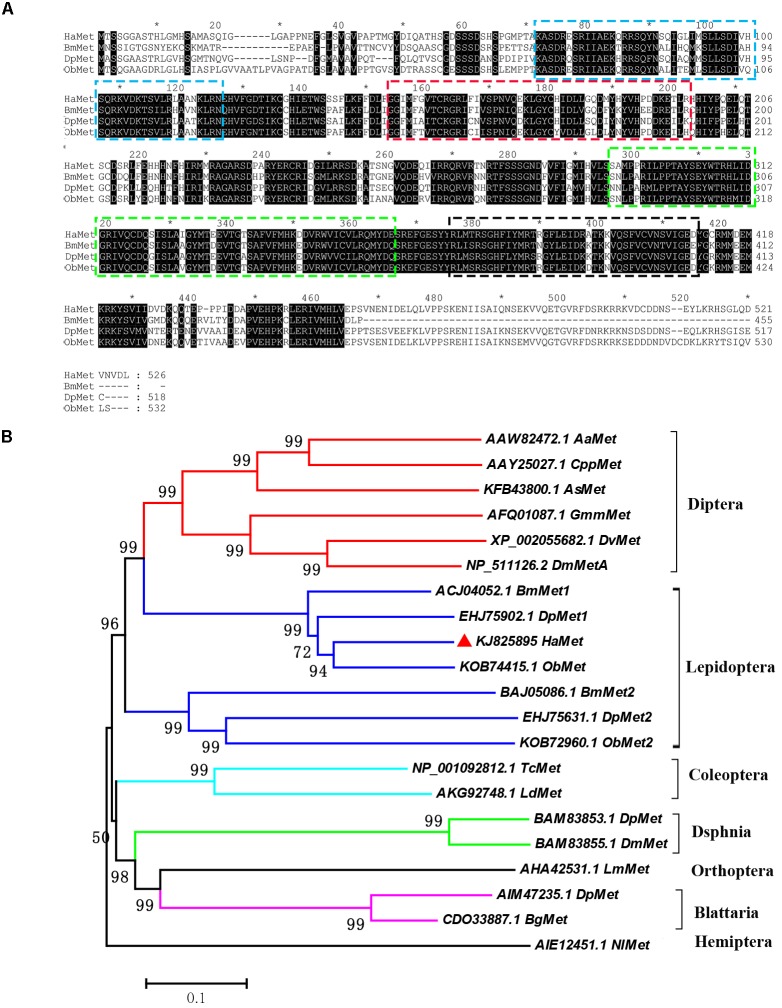
Sequence alignment of *HaMet* with its homologs **(A)** from *Bombyx mori* (*BmMet*, Accession No. ACJ04052.1), *Danaus plexippus* (*DpMet*, Accession No. EHJ75902.1), and *Operophtera brumata* (*ObMet*, Accession No. KOB7445.1). Domains: bHLH (Blue), PAS-A (Red), PAS-B (Green), and PAC (Black) are boxed. **(B)** Phylogenetic analysis of Met homologs from *Diptera*, *Lepidoptera, Coleoptera, Dsphnia*, *Orthoptera*, *Blattaria*, and *Hemiptera* species. Bootstrap values (%) were marked above the tree branches.

The predicted protein sequence of *HaMet* was aligned with homologs from *B. mori*, *Danaus plexippus*, and *Operophtera brumata*. The result showed that the HaMet protein exhibited typical bHLH, PAS-A, PAS-B, and PAC (PAS C terminal motif) domains (Figure 1A). Phylogenetic analysis was constructed on the basis of the protein sequences of Met homologs from various insect species. As expected, *HaMet* is clustered with Mets from lepidopteran *B. mori*, *D. plexippus*, and *O. brumata*, forming an orthologous group of Met1. The result indicated that they originated from the same ancestors and shared conserved functions. However, *HaMet* appears to be more closely related to the Dipteran homologs than other lepidopteran Met homologs of the Met2 group (Figure 1B).

### Tissue and Temporal Expression of *HaMet* During *H. armigera* Development

**FIGURE 2:**
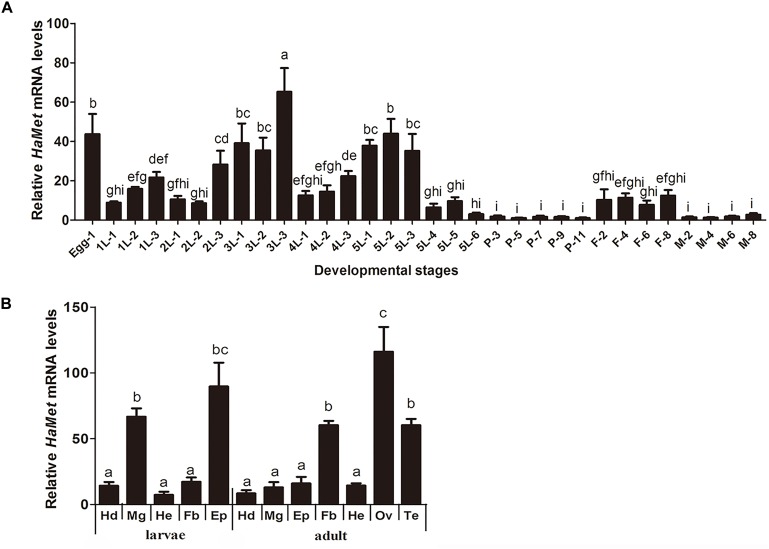
Tissue and temporal expression patterns of *HaMet*. **(A)** Temporal expression of *HaMet* at different developmental stages. The samples are prepared at an interval of 1 day during larval stages and 2 days for pupae and adult. **(B)** Tissue distribution of *HaMet* transcripts in adults and 5th-instar larvae. Hd, head; Mg, midgut; He, head; Fb, fat body; Ep, epidermis; Ov, ovary; Te, testis. The bars represent the average (±SE) of biological repeats. Different letters indicate significant difference between specimens (*P* < 0.05).

The expression pattern of *HaMet* in different stages was determined by qRT-PCR analysis (Figure 2A). The results showed that *HaMet* was detected throughout the entire life cycle, and the transcription level of *HaMet* fluctuated during developmental stages. *HaMet* was highly expressed in the embryonic stage (egg), sharply increased before each molting in the initial four larval instars, decreased at the end of the 5th instar, and maintained at a low level during the pupal stage. In the adult stage, *HaMet* had a notably higher expression in females than in males (Figure 2A).

The tissue expression profiles of *HaMet* in larvae and adults were also examined. In the larval stage, *HaMet* was highly expressed in epidermis and midgut, whereas low expression was observed in head, fat body, and hemolymph. In adults, the *HaMet* transcripts were maximally expressed in the ovary, testis, and fat body, and relatively low expression levels were found in other tissues (Figure 2B).

### Immunostaining of HaMet Protein in Midgut and Ovary

In the preparation for HaMet localization, the specificity of HaMet antiserum was verified by western blot analysis. The result showed that the staining of crude ovarian extracts with HaMet antiserum showed a single band at approximately 60 kDa which is of the predicted size of *HaMet* protein (Supplementary Figure S1).

**FIGURE 3:**
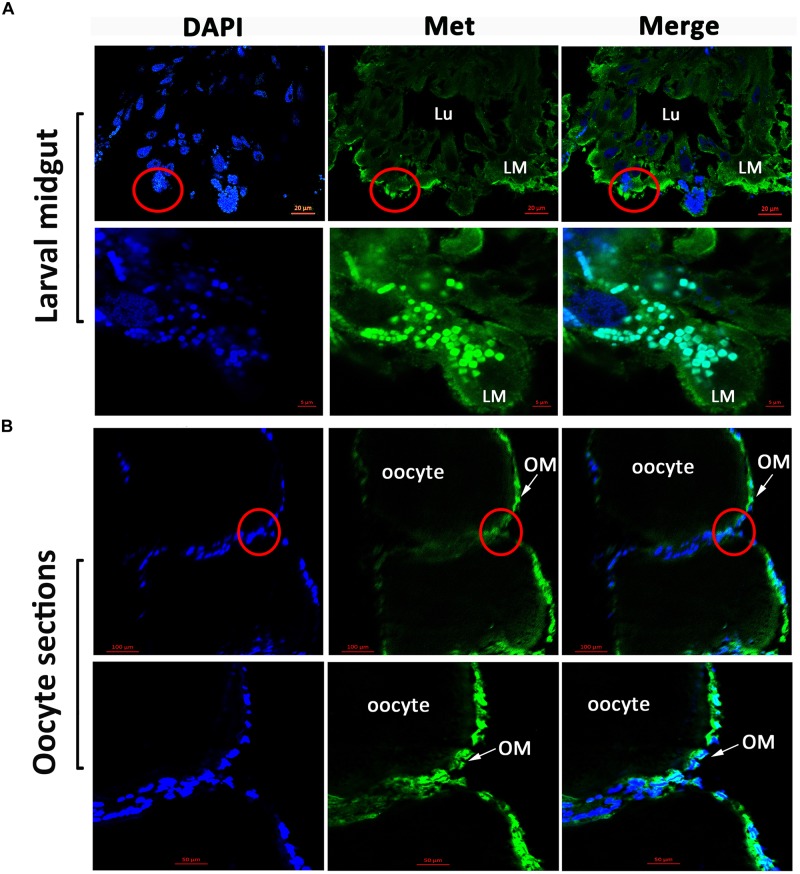
Immunofluorescence observation of HaMet protein. **(A)** Immunostaining of HaMet (green) in larval midgut. HaMet was stained in the longitudinal and circular muscle layers. **(B)** Immunostaining of *HaMet* (green) in oocyte sections of ovary tissue from female adult. DAPI indicates the cell nucleus (blue), and the merge is the overlapped images of green and blue. Lu, midgut lumen; LM, larval midgut; OM, oolemma.

Immunofluorescence microscopy revealed that HaMet was localized in the longitudinal and circular muscle layers of the midgut in larvae (Figure 3A). In contrast in the ovary of female adults, the labeled cells were highly enriched in the oocyte membrane, and HaMet was localized in the cell nucleus (Figure 3B and Supplementary Figure S2).

### Knockdown of *HaMet* Induced Larval Metamorphosis

**FIGURE 4:**
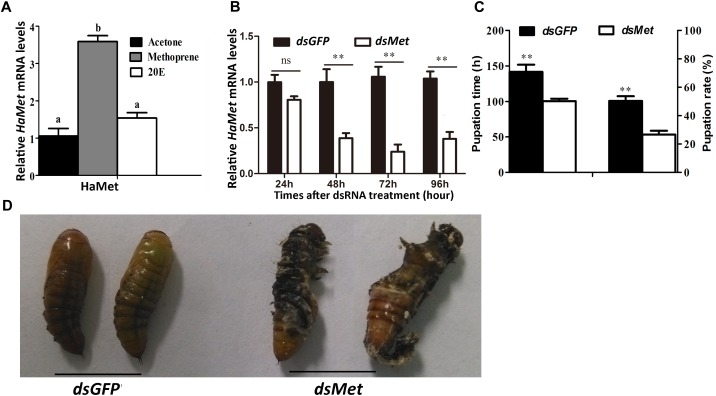
Knockdown of *HaMet* in 5th-instar *H. armigera* larvae. **(A)** The induction of *HaMet* expression by Methoprene (JH analog) or 20E. **(B)** The expression levels of HaMet in larval midgut after ds*GFP* or ds*HaMet* treatment examined at 24, 48, 72, and 96 h after treatment. **(C)** Statistical analysis of the pupation time and pupation rate after *HaMet* knockdown (30 larvae, three replicates). **(D)** The abnormal pupation after *HaMet* knockdown. Error bars indicate SE, ^∗^denotes *P* < 0.05, ^∗∗^denotes *P* < 0.01 (Student’s *t*-test). Different letters indicate significant difference between specimens (*P* < 0.05). ns, indicates no significant difference (*P* > 0.05).

The metamorphic action of larval-pupal transition was regulated by JH under the control of ecdysteroids. To examine whether *HaMet* was regulated by JH or 20E, methoprene (JH analog) and 20E were applied to the 5th-instar larvae. The results showed that *HaMet* expression was upregulated by 3.78 times after methoprene treatment; however, *HaMet* expression was not induced by 20E or acetone (Figure 4A). These results indicated that JH analog (JHA) was the regulator of *HaMet* expression.

The function of HaMet in larval development was further investigated by RNAi of *HaMet* in the 5th-instar larvae. The result showed that 62% of the *HaMet* transcripts were silenced as compared with those in the ds*GFP*-treated group at 48 h postinjection. At 72 h and 96 h post-treatment, the transcription levels of *HaMet* were decreased by 70 and 55% in ds*HaMet*-treated individuals relative to the controls, respectively (Figure 4B).

Caste-differentiation bioassays were used to examine the effect of *HaMet* knockdown in terms of phenotype and development time. The pupation time in ds*GFP*-treated larvae was 48 h longer than that in ds*HaMet*-treated individuals (Figure 4C), indicating that knockdown of HaMet resulted in an early pupation. Besides, phenotypic analysis revealed that ds*HaMet* treatment caused the abnormal pupation in approximately 35% of the treated larvae, such as the persistence of larval-pupal intermediates (Figure 4D). The pupation rate in the ds*HaMet-*treated group declined to 26.68%, which indicated a 47.06% decrease compared with that in control (*P* < 0.01). Taken together, these results demonstrated that HaMet functioned in suppressing larval metamorphosis.

### Met Is Required for Vg Synthesis and Uptake in Female Reproduction

**FIGURE 5:**
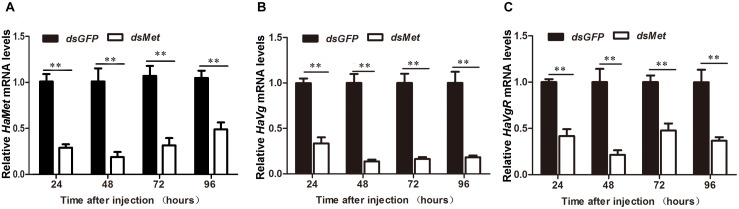
Knockdown of *HaMet* in 1-day-old female adults of *H. armigera*. The relative expression levels of *HaMet* **(A)**, *HaVg* **(B)**, and *HaVgR* **(C)** after ds*GFP* or ds*HaMet* injection examined at 24, 48, 72, and 96 h after the treatment. Error bars indicate SE. ^∗^Denotes *P* < 0.05, ^∗∗^denotes *P* < 0.01 compared with the respective ds*GFP* control (Student’s *t*-test).

In insects, vitellogenin (Vg) is primarily synthesized in fat body to meet the nutrient requirement for egg development. To explore the participation of HaMet in Vg synthesis, we knocked down the expression of *HaMet* in newly emerged female adults, and subsequently examined Vg transcription in fat body of treated females. The results showed that the expressions of *HaMet* in the fat body were reduced by 62.7, 87.6, 60.0, and 43.8% at 24, 48, 72, and 96 h post-treatment, respectively (Figure 5A). Meanwhile, knockdown of *HaMet* caused the significant reduction in Vg expression. As shown in Figure 5B, injection of ds*HaMet* reduced the *HaVg* mRNA levels to 40.5% (24 h post-treatment), 22% (48 h), 28% (72 h), and 30% (96 h) of its normal levels in fat body. Besides, the expressions of *HaVgR* in the ovary were also significantly reduced after *HaMet* knockdown, and an average of 50% decrease in *HaVgR* mRNA levels was observed (Figure 5C).

**FIGURE 6:**
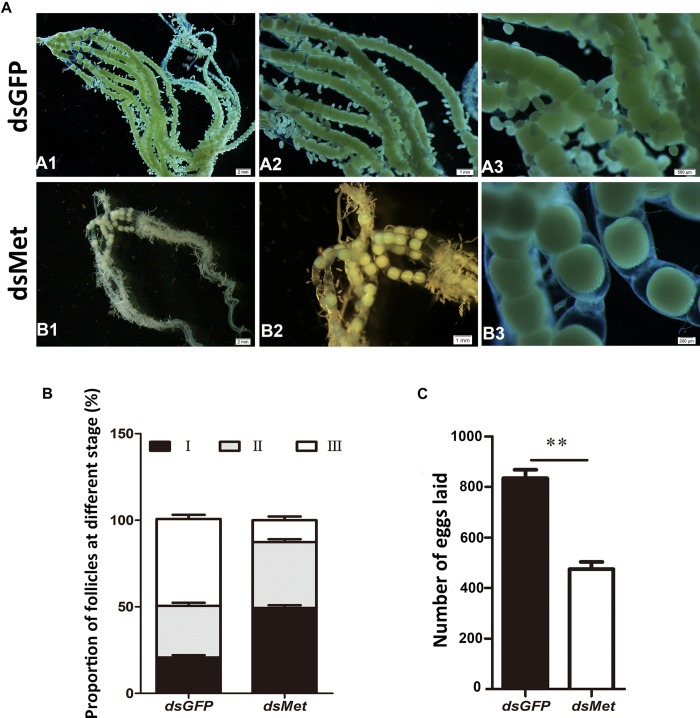
Effects of *HaMet* depletion on oocyte maturation and oviposition in female adults of *H. armigera*. **(A)** The observation of ovarian morphology after *HaMet* knockdown. **A1**, **A2** and **A3** is the upwards-magnifying images of the oocyte sections, and **B1**, **B2** and **B3** is in the same situation. **(B)** Proportion of follicles at different developmental stages (stages I, II, and III) in ovary at 72 h post-treatment. **(C)** Effect on oviposition after *HaMet* knockdown. The number of eggs laid is the sum of daily egg number per female. Error bars indicate SE. ^∗^Denotes *P* < 0.05, ^∗∗^denotes *P* < 0.01 (Student’s *t*-test).

To evaluate the effect of *HaMet* silencing on oviposition and ovary development, the ovaries from both the treated and control female adults were observed 3 days after the treatment, and the number of eggs was recorded. The ovarian morphology showed that knockdown of *HaMet* resulted in an apparent decrease in yolk protein deposition, causing a small degree of yolk uptake in oocytes (Figure 6A). The number of follicles, particularly the mature follicles, was significantly fewer in *HaMet*-silenced moths than that in controls (Figures 6A,B) (*P* < 0.001). Moreover, ds*HaMet*-treated moths exhibited a 44% decline in oviposition compared with those treated with ds*GFP* (*P* < 0.001) (Figure 6C).

**FIGURE 7:**
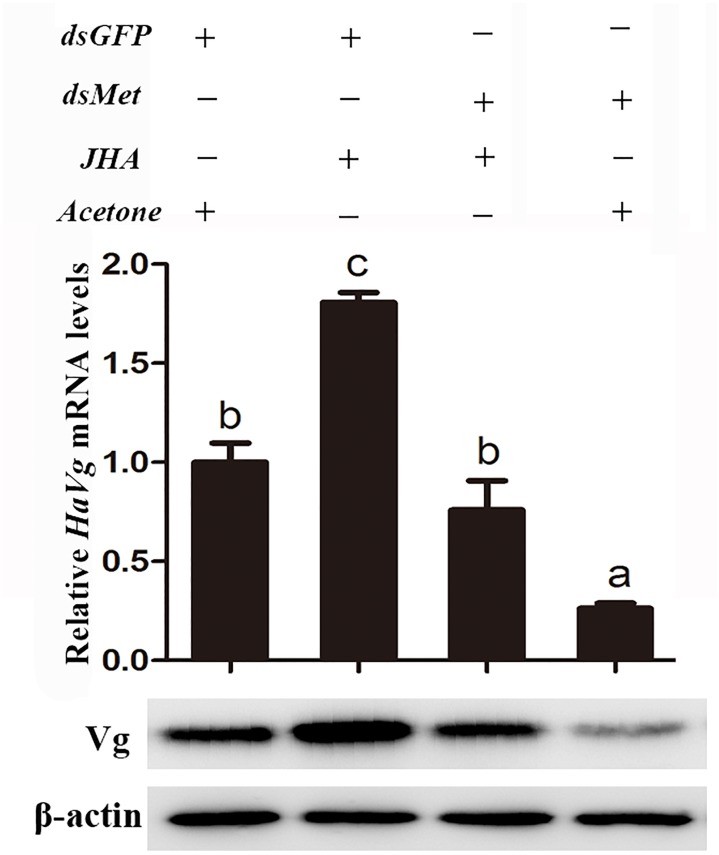
Relative mRNA and protein levels of HaVg in the fat body of ds*GFP* or ds*HaMet*-treated females ingested with JHA. The newly emerged female moths were injected with ds*GFP* or ds*HaMet*, and reared for 1 day. After that, 20E, methoprene, or acetone was topically applied to the treated individuals. The error bars indicate SE. Different letters indicate significant difference between specimens (*P* < 0.05).

To further explore the role of Met in JH-mediated vitellogenesis, JHA was applied to the *HaMet*-silenced moths (Figure 7). The results showed that the expression of *HaVg* and the content of Vg protein failed to recover in the *HaMet*-silenced moths, indicating that the capacity of methoprene to induce *HaVg* expression in the fat body was completely blocked by *HaMet* knockdown.

## Discussion

In insects, JH has primary roles in repressing metamorphosis and stimulating several aspects of reproduction (Riddiford, 2008). Multiple studies have confirmed that Met is an essential receptor in the JH signaling pathway (Konopova and Jindra, 2007). Here, we identified and characterized *HaMet* from *H. armigera*. Similar to other known Met genes, *HaMet* contains an HLH structure with two variably spaced PAS domains (A and B), and a hallmark of the bHLH-PAS protein family (Ashok et al., 1998). Phylogenetic analysis revealed that HaMet was clustered with three lepidopteran Mets into a separate clade (Met1 group) that was closely related to Dipteran Mets.

The temporal expression pattern showed that *HaMet* was sharply increased before each molting in the larval stage, and maintained low level during the pupal stage. This stage-specific expression profile is overall consistent with the changes of the *in vivo* JH titer in larvae (Zhang et al., 2017), which supports the previous findings showing that JH exerts its anti-metamorphic effect through its receptor Met (Jindra et al., 2013; Zhao et al., 2014). The similar expression pattern of Met was reported in *T. castaneum* (Parthasarathy et al., 2008). For tissue expression, *HaMet* is highly transcribed in larval midgut, and immunolocalization confirms the distribution of HaMet protein in different layers of longitudinal and circular muscle cells in larval midgut. Coincidentally, the previous study in *H. armigera* has documented that Met depletion could repress the suppressive effect of JH on midgut remodeling (Zhao et al., 2014). Similarly, in *D. melanogaster*, Met protein is present in the midgut imaginal cells during larval-pupal transition (Pursley et al., 2000), and the overexpression of Met leads to the precocious and enhanced programmed cell death (Liu et al., 2009). Interestingly, Rahman et al. (2017) described that JHs were also synthesized by the gut in adult *Drosophila*, and this gut-specific JH activity is synthesized by and acts on the intestinal stem cell and enteroblast populations, regulating their survival and cellular growth through the JH receptors Gce/Met. Consequently, we presume that the midgut-abundant HaMet acts to promote tissue remodeling during the larval-pupal transition in *H. armigera*. However, this function may not be applicable to all insects. In *T. castaneum*, the depletion of *TcMet* did not completely block the remodeling of midgut tissue (Parthasarathy et al., 2008). Therefore, how Met acts in midgut remodeling and whether Met alone is sufficient to promote the midgut metamorphosis needs further study.

Many studies have documented that JH inhibits the larval-pupal transition via Met and the downstream transcription factor Kr-h1 in both holometabolous and hemimetabolous species (Hiruma and Riddiford, 2010; Riddiford, 2012; Jindra et al., 2013; Zhao et al., 2014). Our present study showed that depletion of *HaMet* significantly reduced the pupation time, which supported its function of anti-metamorphosis. Similarly, disruption of *Met* by RNAi led to precocious metamorphosis in the linden bug *Pyrrhocoris apterus* (Konopova et al., 2011). Moreover, in *T. castaneum*, depletion of *TcMet* at the end of the last larval instar resulted in the premature upregulation of the adult-specifier factor TcE93, and led to a direct transformation from larva to the adult form, bypassing the pupal stage (Ureña et al., 2016). Our RNAi study showed that *HaMet* knockdown caused the malformation individuals with the complex characters of larvae and pupae, and the deficiency in pupation was largely attributed to the fact that the treated individuals were incapable of fully forming puparium. Other studies in *T. castaneum* and *B. mori* reported that *Met* depletion in the late larval stage induced a certain level of mortality and complications in ecdysis (Konopova and Jindra, 2007; Parthasarathy et al., 2008; Guo et al., 2012). We presume that the early pupation or the malformation occurring during larval-pupal transition may be attributed to the metabolic deficiencies caused by the Insulin signaling pathway. Actually, the involvement of Met in the insulin signaling pathway has been reported in *T. castaneum* and *Blattella germanica*, where *Met* depletion impaired the expression of insulin-like-peptides (Sheng et al., 2011; Lozano and Belles, 2014).

In addition to its role during metamorphosis, JH also plays a primary role in regulating Vg expression in fat body, and is crucial for the maintenance of follicle patency, uptake of Vg, and choriogenesis (Ramaswamy et al., 1997). The previous study in *H. armigera* has reported the tight correlation between the JH titer and ovariole development (Zhang et al., 2016a). The expression of Vg, the major yolk protein in oocyte, is regulated by JH via its receptor Met, which has been documented in *A. aegypti* (Raikhel et al., 2005; Zou et al., 2013), *P. apterus* (Smykal et al., 2014), and *T. castaneum* (Parthasarathy et al., 2010). Other studies in *Cimex lectularius* (Gujar and Palli, 2016) and *Nilaparvata lugens* (Lin et al., 2015) also confirmed that Met was required for *Vg* expression and ovarian development. Our RNAi experiment revealed that knockdown of *HaMet* resulted in the significant decline of *Vg* expression in the fat body, the reduced expression of *VgR* in the ovary, and the atrophied ovaries with less yolk protein deposition. In other words, the declined transcription of *Vg* from the *HaMet*-depleted fat body blocked the Vg synthesis. Meanwhile, the depleted *VgR* expression in the ovary blocked the uptake of yolk proteins, both of which caused the atrophied ovaries and ultimately impaired female fecundity. Interestingly, the cellular immunolocalization revealed that HaMet was abundantly expressed in the nucleus of oocyte membrane, which corroborated the hypothesis that HaMet acted as the JH-nuclear receptor regulating the uptake of yolk protein during the oocyte formation. Indeed, oogenesis involves the internalization of proteins and lipids from the circulating hemolymph and this internalization process is thought to be mediated in large part by the membrane-associated receptors, and the immunoreactivity showed that most *B. mori* VgRs are localized in the inner oocyte membrane (Han et al., 2017). Taken together, we presume that the abundant expression of HaMet at oolemma is involved in Vg uptake by oocytes.

Juvenile hormone has been proved to play a major role in inducing vitellogenesis in many hemimetabolous insects (Glinka and Wyatt, 1996; Comas et al., 2001). In our study, JHA application induces *Vg* expression in the control group; however, JHA application failed to recover *Vg* expression in the *HaMet*-depleted moths. These results correspond to data from locusts and *P. apterus*, where methoprene treatment on *Met*-depleted individuals failed to restore the defective phenotypes (Smykal et al., 2014; Song et al., 2014). Taken together, these studies indicated that Met mediated the induction of Vg expression in a JH-dependent manner.

The cotton bollworm, *H. armigera*, is a major agricultural pest worldwide. Transgenic crops that produce *Bacillus thuringiensis* (Bt) Cry toxins have become an important tool against this pest. Currently, to counter the increasing pest resistance to transgenic cotton-expressing Bt toxin, plant-mediated RNAi provides a new strategy. Transgenic plants producing dsRNA targeting the crucial genes in insect reproduction and metamorphosis were designed to control agricultural pests (Xiong et al., 2013; Tian et al., 2015; Luo et al., 2017). Our results help to unveil the complex roles of HaMet in insect development and reproduction, and highlight Met as a target for suppression of lepidopteran pests.

## Author Contributions

GL, LM, and WZ conceived and designed the experimental plan. LM, WZ, CL, LC, and YX performed the experiments. LM, WZ, and HX processed and analyzed the data. GL, LM, WZ, and HX wrote and edited the manuscript.

## Conflict of Interest Statement

The authors declare that the research was conducted in the absence of any commercial or financial relationships that could be construed as a potential conflict of interest.

## Footnotes

1 http://www.expasy.org

2 http://smart.emblheidelberg.de/

## Supplementary Material

Figure S1 Western blot analysis of the specificity of HaMet antibody.

Figure S2 Immunocytochemical localization of *HaMet* protein in oocyte section using colloidal gold labeling. Black spots represent the immunostained *HaMet* protein. **(A)** The negative control used the serum supernatant from an uninfected healthy rabbit as the secondary antibody in immunochemistry and **(B)** the enlarged image of oocytes section. **(C)** Cross section through an oocyte shows the strongly immunostained *HaMet* in oolemma. **(D)** Enlarged images of oocyte section reveal the heavy labeling of *HaMet* protein in the oolemma (arrow). The secondary antibody was anti-rabbit IgG conjugated with 10 nm colloidal gold granules at a dilution of 1:20.

Table S1 Primers used in the experiment.
