# Supplementary figures and images for: Methoprene-Tolerant (Met) Is Indispensable for Larval Metamorphosis and Female Reproduction in the Cotton Bollworm Helicoverpa armigera
Source: Front Physiol. 2018 Nov 15;9:1601. doi: 10.3389/fphys.2018.01601 (PMC6249418; doi:10.3389/fphys.2018.01601)

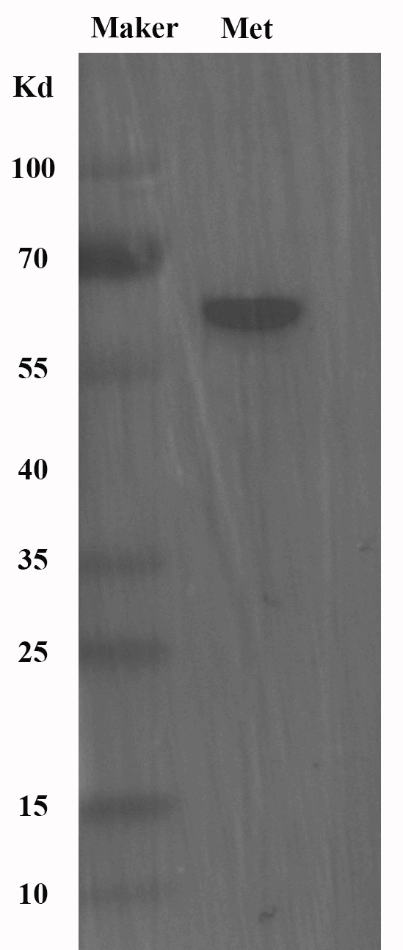

Supplement: Figure S1 — Western blot analysis of the specificity of HaMet antibody. [file Image_1.TIF]

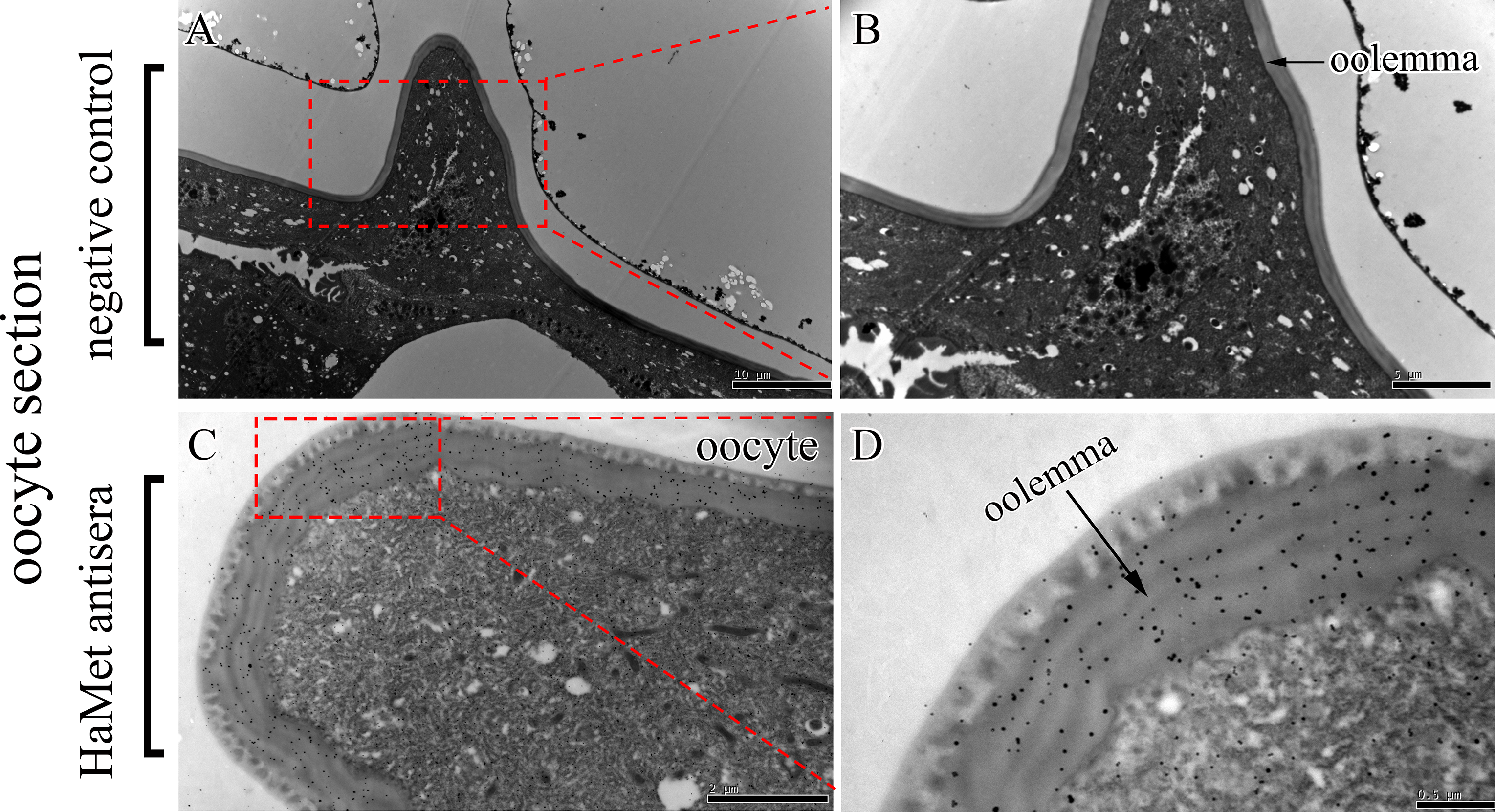

Supplement: Figure S2 — Immunocytochemical localization of HaMet protein in oocyte section using colloidal gold labeling. Black spots represent the immunostained HaMet protein. (A) The negative control used the serum supernatant from an uninfected healthy rabbit as the secondary antibody in immunochemistry and (B) the enlarged image of oocytes section. (C) Cross section through an oocyte shows the strongly immunostained HaMet in oolemma. (D) Enlarged images of oocyte section reveal the heavy labeling of HaMet protein in the oolemma (arrow). The secondary antibody was anti-rabbit IgG conjugated with 10 nm colloidal gold granules at a dilution of 1:20. [file Image_2.TIF]
