# Supplementary material for: Methoprene-Tolerant (Met) Is Indispensable for Larval Metamorphosis and Female Reproduction in the Cotton Bollworm Helicoverpa armigera
Source: Front Physiol. 2018 Nov 15;9:1601. doi: 10.3389/fphys.2018.01601 (PMC6249418; doi:10.3389/fphys.2018.01601)
Supplement: Table S1 — Primers used in the experiment. [file Table_1.DOCX]

**Table S1.** Primers used in the experiment.

| Primer name | Forward | Reverse |
| --- | --- | --- |
| Met | AGCGTGCAGCAGTATCGGT | ACATTAACATCTTGCAACCCACTAT |
| Met-5’RACE | CCGAGATGCGTACTTGCTCCGCCTGAAG | |
| Met-3’RACE | GGAAGAGGCGAAAGGTGGACTGTGATGAC | |
| UPM short  UPM long | CTAATACGACTCACTATAGGGC  CTAATACGACTCACTATAGGGCAAGCAGTGGTATCAACGCAGAGT | |
| M13 | TGTAAAACGACGGCCAGT | CAGGAAACAGCTATGACC |
| RNAi  Meti | TAATACGACTCACTATAGGG  CAATGCCACCTCGAATTCT | TAATACGACTCACTATAGGGTT  ACAGATCAACATTAACATCTTGCA |
| GFPi | TAATACGACTCACTATAGGGG  CAACATACGGAAAACTTACC | TAATACGACTCACTATAGGG  TGTGTGGACAGGTAATGGTTG |
| qRT-PCR |  |  |
| Actin  EF-1α  Met  HaVg  HaVgR | GCGACATCAAGGAGAAGCTG  GGGCAAGGAAAAGATTCACA  CTATCCAGTGCAATGCCACC  CCTTGGTGCCCTACTCTCAA  CCTGCAACAACAGCACATGTT | CGTCGCACTTCATGATGGAG  GGCCTCCTTCTCGAACTTCT  CCCAGCGGACATCATCTTTG  TGGGTCAAAGCGATGTCTCT  AAGGGACAGACGCATTGCTT |
